# BET bromodomain inhibitors synergize with ATR inhibitors in melanoma

**DOI:** 10.1038/cddis.2017.383

**Published:** 2017-08-10

**Authors:** Somsundar Veppil Muralidharan, Berglind Osk Einarsdottir, Joydeep Bhadury, Mattias F Lindberg, Jin Wu, Eric Campeau, Roger Olofsson Bagge, Ulrika Stierner, Lars Ny, Lisa M Nilsson, Jonas A Nilsson

**Affiliations:** 1 Sahlgrenska Cancer Center, Department of Surgery or University Hospital, Gothenburg, Sweden; 2 The Institute of Medical Science, Division of Stem Cell Therapy, The University of Tokyo, Tokyo, Japan; 3 Zenith Epigenetics Ltd, Calgary, Canada; 4 Department of Oncology, Institute of Clinical Sciences, Sahlgrenska Academy, University of Gothenburg and The Sahlgrenska University Hospital, Gothenburg, Sweden

## Abstract

Metastatic malignant melanoma continues to be a challenging disease despite clinical translation of the comprehensive understanding of driver mutations and how melanoma cells evade immune attack. In Myc-driven lymphoma, efficacy of epigenetic inhibitors of the bromodomain and extra-terminal domain (BET) family of bromodomain proteins can be enhanced by combination therapy with inhibitors of the DNA damage response kinase ATR. Whether this combination is active in solid malignancies like melanoma, and how it relates to immune therapy, has not previously investigated. To test efficacy and molecular consequences of combination therapies cultured melanoma cells were used. To assess tumor responses to therapies *in vivo* we use patient-derived xenografts and B6 mice transplanted with B16F10 melanoma cells. Concomitant inhibition of BET proteins and ATR of cultured melanoma cells resulted in similar effects as recently shown in lymphoma, such as induction of apoptosis and p62, implicated in autophagy, senescence-associated secretory pathway and ER stress. *In vivo*, apoptosis and suppression of subcutaneous growth of patient-derived melanoma and B16F10 cells were observed. Our data suggest that ATRI/BETI combination therapies are effective in melanoma.

Malignant melanoma (MM) is potentially curable if diagnosed early but if the disease becomes metastatic it often is fatal. Recent advances in the molecular and immunological characterization of the disease have generated new promising avenues of therapeutic intervention.^1^ First, the discovery of the mutated *BRAF* oncogene^2^ enabled the development of targeted kinase inhibitors that exhibited remarkable objective response in patients with MM carrying the mutated *BRAF*^V600^ allele.^3, 4^ Additional inhibition of the downstream MAPK pathway using MEK inhibitors has further prolonged overall survival^5^ but in most cases relapses of lethal and therapy resistant clones emerge. Multiple resistance pathways have been found,^6, 7^ suggesting new combination treatments that are tested in various clinical trials. Second, the discovery of immune checkpoints^8^ enabled the development of antibodies directed against CTLA4 and PD-1 (or its ligand PDL1), which show lower response rates but generally more durable responses.^9, 10^ Taken together, these modern treatments have been successful, but to cure or make MM manageable chronic, more and safe drugs are needed.

BET proteins are emerging targets for cancer therapy.^11^ BET proteins regulate transcription and appear to be utilized during cancer progression to epigenetically reprogram both blood and solid cancers.^12^ Small-molecule inhibitors of BET proteins are in clinical trials but preclinical models already suggest that combination therapies will be needed to further the efficacy of BET inhibitors (BETIs). Indeed, we and other investigators have recently demonstrated that inhibitors targeting cell-signaling molecules, the proteasome, components of the DNA damage response and HDAC synergize with BETIs to kill B-cell malignancies.^13, 14, 15, 16, 17, 18, 19^ HDAC and BETI combination treatment is also effective in melanoma^20^ but whether any of the other therapies would be effective is not known.

Ataxia-telangiectasia and Rad3-related (ATR) is a kinase that belongs to the PI3-kinase-like family, which also includes PI3K, mTOR, ATM and DNA-PK. ATR has a critical role in the regulation of replication and is activated by replication fork stalling. Known causes of stalling include UV-induced DNA damage and nucleotide deprivation but also excess replication fork firing exerted by oncogenic replication stress.^21^ When ATR is activated it phosphorylates the checkpoint kinase Chk1 that work together with ATR to phosphorylate components in the replication machinery to inhibit further replication. Hence, inhibition of ATR or Chk1 is detrimental to cells experiencing replication fork problems such as cancer cells expressing high levels of the *MYC* oncogene.^22, 23, 24, 25^

Both BETIs and Chk1 inhibitors have previously been shown to have efficacy in cultured melanoma cells and Chk1 has even been suggested to be essential for the melanocytic lineage.^26^ We have demonstrated that Myc-induced lymphoma cells undergoing replication stress, because of ATR inhibition, are sensitive to BETIs.^19^ Here we wish to investigate whether or not this finding can be extended to solid cancers. By using cultured melanoma cells, patient-derived xenografts (PDXs) and syngenic transplant models we show that the therapeutic combination targeting of ATR and BET proteins is effective in melanoma.

## Results and Discussion

### BET bromodomain inhibitors synergize with ATR inhibitors to induce apoptosis, and senescence-associated secretory pathway in melanoma

**Figure 1:**
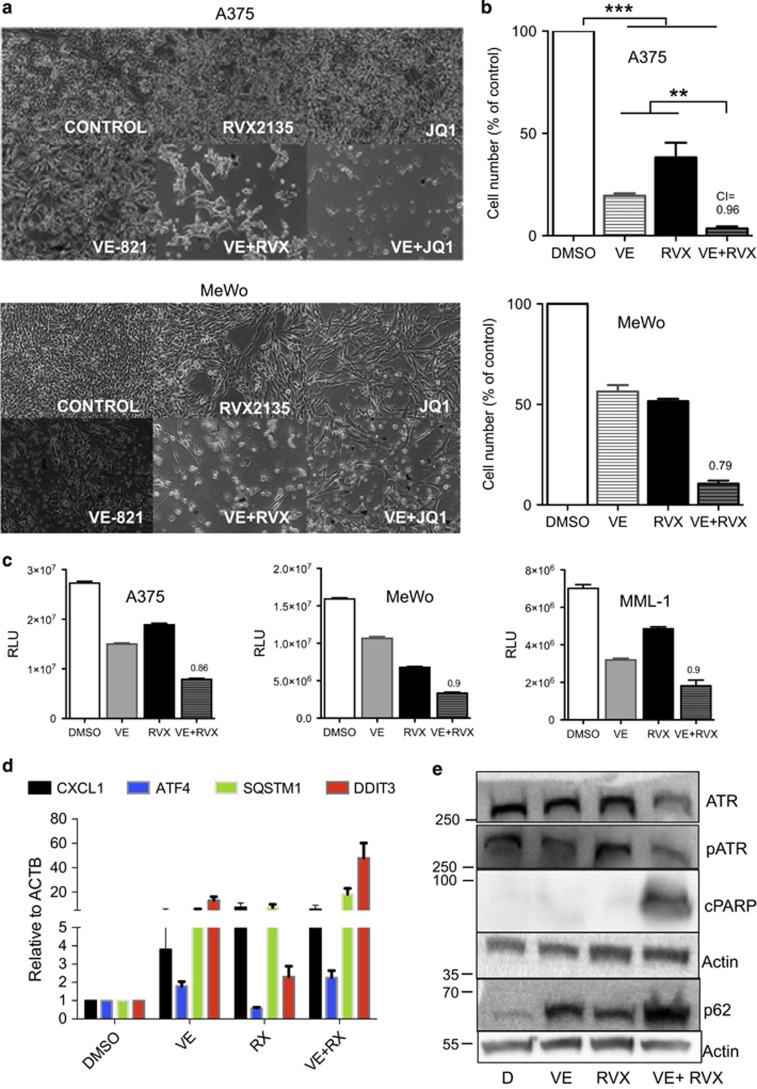
ATRIs synergize with BETi to kill melanoma cells and induce SASP/ER stress. (**a** and **b**) A375 cells (*BRAF*^V600E^) and MeWo cells (*NF1*^−/−^) were treated with vehicle (0.1% DMSO), 10 *μ*M VE821 (VE; AXON Medchem, Groningen, The Netherlands), 1 *μ*M JQ1 (Cayman Chemicals, Ann Arbor, MI, USA), 10 *μ*M RVX2135 (RVX) or indicated combinations. The experiments were repeated twice in biological triplicates. Cells were imaged in a light microscope (**a**) or counted in a hemocytometer (**b**). (**c**) A375 and MeWo cells were cultured in the presence of vehicle (DMSO), 10 *μ*M of RVX2135 (RVX) and/or the ATRI VE821 (VE; 10 *μ*M) for 48 h, and were assayed for viability with CellTiter Glo. Value to achieve synergy is shown with a dotted line. (**d**) A375 cells treated with vehicle, 10 *μ*M VE821, 10 *μ*M RVX2135 or both VE821/RVX2135 were analyzed by qRT-PCR for indicated genes. (**e**) A375 cells treated as described above and analyzed by western blot analysis using indicated antibodies

Melanoma cells are sensitive to the BETIs JQ1, iBET-151 and RVX2135 (Supplementary Figure S1 and shown by others^15, 27, 28^). To assess the therapeutic effect of combined inhibition of ATR kinase and BET protein we cultured the melanoma cell lines A375 and MeWo in the presence of the ATR inhibitor (ATRI) VE821 and/or RVX2135.^15, 19^ Both compounds were antiproliferative as assessed by microscopy, CellTiter Glo (Promega, Madison, WI, USA) measurements and cell counts (Figures 1a–c). Combining the two generated profound effects on the viability of the cells and combination index calculations showed that the compounds synergized (Figures 1b and c).

In our previous study we showed that ATRI/BETI combination therapy of B-cell lymphoma resulted in a gene expression profile resembling that of senescence-associated secretory pathway (SASP) and ER stress.^19^ Examining the melanoma cells treated with ATRIs/BETIs that had not undergone apoptosis it was evident that the cells had large vacuoles or vesicles in their cytoplasm; this was mostly evident in combination-treated cells but also seen in ATRI-treated cells (Figure 1a). We therefore performed qRT-PCR and western blot analyses on A375 cells and probed for components of SASP/ER stress that we had found deregulated in lymphoma cells responding to the combination treatment. Indeed, the mRNA encoding the SASP cytokine Cxcl1, the ER stress master regulator ATF4 and the SASP/ER stress regulators SQSTM/p62 and DDIT3/CHOP were all induced by ATR and in combination-treated cells albeit not in a synergistic manner (Figure 1d). Western blot analysis confirmed that the combination treatment synergistically induced p62 (Figure 1e; Supplementary Figures S2A–C). The apoptotic marker cleaved PARP was induced in A375 cells and MeWo cells (Figure 1e), but Bim and the ER stress regulators CHOP and ATF4 (Supplementary Figures S2C and D) were not. As the ER stress inducer tunicamycin potently induced CHOP and ATF4 (Supplementary Figure S2C) it is possible that BETi/ATRi change the rate of translation or induce protein turnover, which would explain the discordance between the RNA and protein levels of CHOP and ATF4. Interestingly, GATA4, a component of SASP was downregulated (Supplementary Figure S2D), which could explain why a full SASP phenotype was absent. Taken together our data suggest that cultured melanoma cells are sensitive to ATRI/BETI combination treatment and hence that this new treatment is effective in more settings than Myc-induced lymphoma.^19^

### ATRI and BET combination treatment can induce apoptosis, SASP and ER stress in melanoma tumors in mice

**Figure 2:**
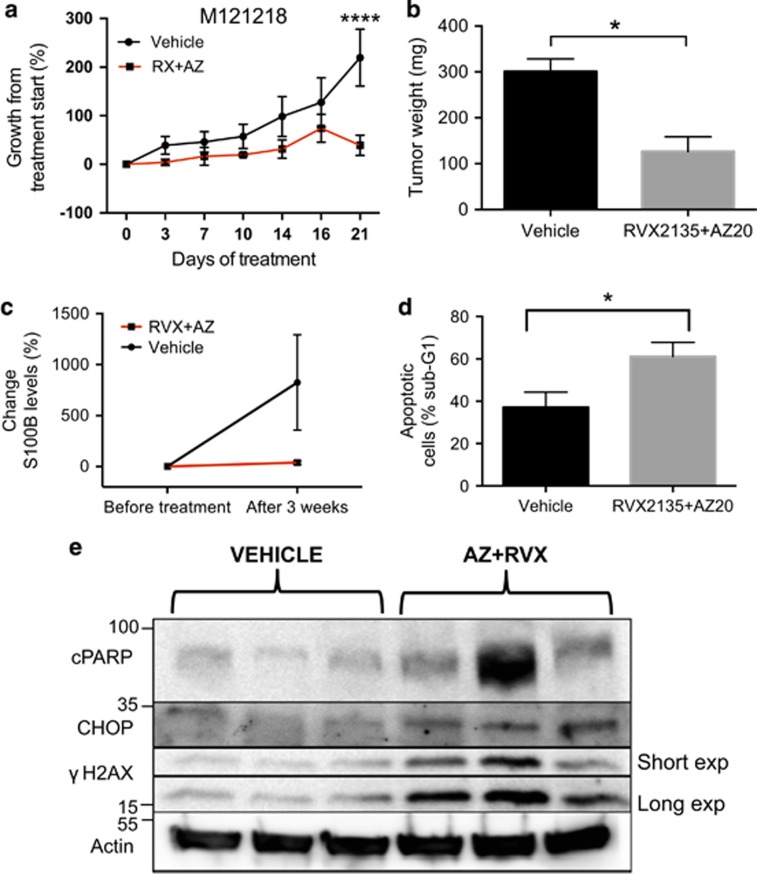
ATRIs synergize with BETi to kill melanoma cells. (**a**) The melanoma PDX model M121218 was initiated by thawing a stock of cryopreserved melanoma tumor cells,^30^ and injecting the cells subcutaneously into the flank of 10 immunocompromised NOD/SCID/IL2R*γ* mice (Taconic). Tumor sizes were measured bi-weekly using an caliper. When the tumors reached 75–100 mm^3^ 5 mice each were randomized to receive either oral and i.p. vehicle, or oral RVX2135 at 75 mg/kg b.i.d. and i.p. injection of AZ20 (MedChemExpress, Princeton, NJ, USA) at 50 mg/kg q.d. for 5 days a week. (**b**) Four hours after the last dose, tumors were excised and weighed. (**c**) A blood sample was drawn from the saphenous vein of all mice before treatment and after 3 weeks of treatment. Plasma was isolated and used to determine the level of the melanoma marker S100B using an ELISA kit from Abcam (Elisa kit from Abnova, Taipei City, Taiwan). (**d**) Single cells were derived by trypsinization of excised tumors from vehicle-treated or combination-treated mice. The cells were lysed and their nuclei were labeled with 7-AAD. Sub-G1 content (apoptosis) was measured by flow cytometry. (**e**) Tumor pieces from M121218 PDXs treated with vehicle or the RVX2135/AZ20 combination treatment were subjected to western blot analysis

Cultured melanoma cells are grown in very different conditions than melanoma cells in patients or in mice.^29^ We recently developed a platform of highly characterized PDX models.^30^ To test whether ATRI/BETI would work in a PDX model end we had to use the bioavailable ATRI AZ20, as VE821 is not bioavailable *in vivo*. In PDX model M121218 we observed a robust reduction in subcutaneous growth and tumor size (Figures 2a and b), a reduced serum level of the melanoma marker S100B (Figure 2c), and a marked increase of apoptotic cells in the excised tumor (Figure 2d). To investigate whether apoptosis and SASP/ER stress was induced by ATRI/BETI treatment also *in vivo* we subjected excised tumors to western blot analysis (Figure 2e). As seen *in vitro* and in lymphoma^19^ there was an induction of cleaved PARP, indicating apoptosis, increased levels of SASP/ER stress marker DDIT3/CHOP and increased levels of phosphorylated H2Ax (*γ*H2AX), a marker of double-stranded DNA damage that often follows ATR/Chk1 inhibition.^31, 32^

**Figure 3:**
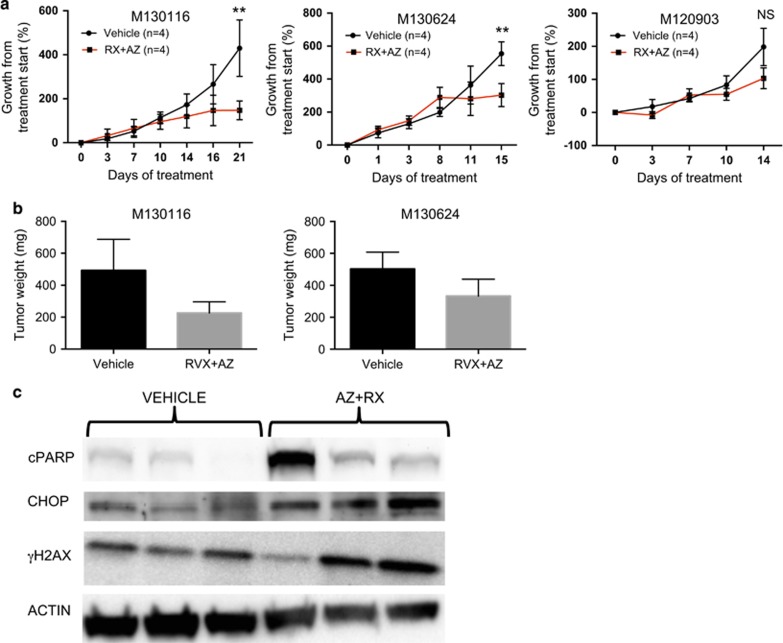
Combined ATR and BET inhibition reduces growth of patient-derived melanoma tumorgrafts in mice. (**a**) Growth of three melanoma PDX models, originally developed from a biopsies of patients’ metastases, transplanted subcutaneously onto the flank of NOG mice (*n=*4 per treatment group). When the tumors reached 75–100 mm^3^ they were randomized to receive either oral vehicle or 75 mg/kg RVX2135 b.i.d. and an intraperitoneal injection of vehicle or 50 mg/kg AZ20 q.d. five times per week. After the first week of treatment PDX model M120903 exhibited sings of treatment-induced distress so the dose of AZ20 was reduced to 25 mg/kg q.d. for all mice of all PDX models subsequently treated to avoid complications. (**b**) Four hours after the last dose, tumors were excised and weighed from the indicated PDX models. The tumor texture of M120903 was very loose preventing accurate weighing. (**c**) Tumor pieces from M130116 PDXs treated with the vehicle or the RVX2135/AZ20 combination treatment were subjected to western blot analysis

To investigate whether tumors from other patients would be sensitive to the ATRI/BETI combination therapy we treated three other PDX models. In two of these models models, the combination treatment blocked growth resulting in smaller tumors and induction of apoptosis (Figures 3a–c). In the fourth model, derived from a lymph node metastasis of patient M120903, the initiation of treatment resulted in adverse effects and drug-related death and the need to decrease the dose of AZ20 (Figure 3a). This is suggestive of tumor lysis syndrome akin to what was observed in lymphoma-bearing mice with large tumors treated with the ATRI/BETI combination treatment.^19^

**Figure 4:**
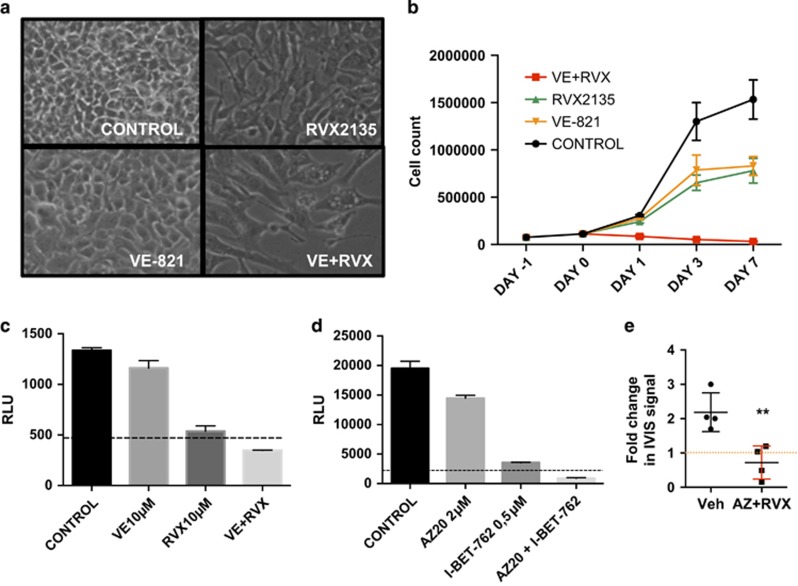
ATRI/BETI combination treatment kills B16 melanoma cells *in vitro* and *in vivo*. (**a**) B16F10-luciferase cells were cultured in RPMI-1640 emented with 10% fetal bovine serum and antibiotics. They were treated with vehicle (0.1% DMSO), 10 *μ*M VE821 (VE), 10 *μ*M RVX2135 (RVX) or indicated combinations. The experiments were repeated twice in biological triplicates. Cells were imaged in a light microscope (**a**) or counted in a hemocytometer (**b**). (**c**) B16F10-luciferase cells were cultured in the presence of vehicle (DMSO), 10 *μ*M of RVX2135 (RVX) and/or the ATRI VE821 (VE; 10 *μ*M) for 48 h and were assayed for viability by adding luciferin (Perkin-Elmer) to a final concentration of 100 *μ*g/ml. Value to achieve synergy is shown with a dotted line. (**d**) B16F10-luciferase cells were cultured in the presence of vehicle (DMSO), 0.5 *μ*M of iBET762 and/or 2 *μ*M of the ATRI AZ20 for 48 h and were assayed for viability by adding luciferin (Perkin-Elmer) to a final concentration of 100 *μ*g/ml. Value to achieve synergy is shown with a dotted line. (**e**) Eight 6- to 8-week-old C57BL/6 Albino mice were transplanted subcutaneously with 10^5^ B16F10-luciferase cells. Seven days after transplantation, mice were imaged in an IVIS Lumina III XR machine. After imaging, mice were treated with oral and i.p. vehicle, or oral RVX2135 at 75 mg/kg b.i.d. and i.p. injection of AZ20 at 25 mg/kg for 4 days, followed by imaging again. Shown is the fold change in luciferase signal during treatment (*n=*4 mice per treatment group)

Finally, to test the effect of the combination in a mouse model, which has immune cells we turned to a widely used murine melanoma cell line, B16F10, which can be grown in syngenic C57BL/6 mice. We first cultured B16F10 cells *in vitro* in the presence or absence of ATRI (VE821 or AZ20) and/or BETI (RVX2135 or iBET762). The cells were noticeably sensitive to BETI, less to ATRI but very sensitive to the combination therapy (Figures 4a–d), irrespective of which BETI or ATRI that was used, suggesting on-target effects. Again vacuole-like or lysosome-like structures were evident in the combination-treated cells (Figure 4a), and long-term culture killed the cells, whereas single-treated cells were growth-inhibited (Figures 4b–d). We tested the effect of the ATRI/BETI treatment *in vivo,* by injecting luciferase-expressing B16F10 cells subcutaneously. One week after transplant, mice were imaged and then treatment was commenced. Because the single drugs were insufficient *in vitro* we tested the combination treatment *in vivo*. Reassuringly, treatment with the combination treatment reduced the luciferase signal from the B16F10 (Figure 4e). Taken together our data suggest that melanoma cells from humans and mice are sensitive to ATRI/BETI combination treatment *in vitro* and *in vivo* and hence that this new treatment could be effective in more settings than Myc-induced lymphoma.

To conclude, it is worth noticing that targeted therapies directed against the mutated driver *BRAF* have potent yet short-term effects and do not work in the half-of-all melanomas that lack *BRAF* V600 mutations. Immune therapies have longer effects but fewer patients respond. Therefore, additional therapies targeting the cancer cell’s engine, rather than its driver, is needed. First, an obvious approach is to target the transcription factors deregulated in the cancers, such as MYC. The concept has been validated in many preclinical models^33^ but to date no effective therapy is present in the clinic. Second, other interesting targets are those harnessing the genetic stability – a known cancer cell vulnerability.^34^ Inhibitors of DNA repair proteins, such as PARP,^35^ and checkpoint kinases, such as Chk1 and ATR,^23, 25^ are undergoing clinical development alone or in combination with classical chemotherapy. Third, to target epigenetic readers, writers and erasers is an additional approach capitalizing on the epigenetic changes in cancer cells that have been recognized for decades. Three HDAC inhibitors (HDACI) and two DNA methyltransferase inhibitors are already approved for various malignancies. Several BETI, histone/lysine methyltransferase inhibitors and HAT inhibitors are in various stages of development. Combination therapies are an obvious approach but which therapies to combine, when to combine and how to combine to maximize efficacy and minimize side effects is still unknown. We have identified two different possible combination therapies against Myc-induced cancers, BETI/HDACI^15^ and BETI/ATRI (here and Muralidharan *et al.*^19^). It appears as if the mechanisms are broad, resulting in cell death and large changes in transcriptional output. On the other hand, it remains to be found whether or not there are selective events that are shared between these two potent combination therapies that can be further utilized in design of new therapeutic approaches.

## Materials and methods

### Cell experiments

All cell lines were from Cell Lines Services (Eppelheim, Germany). They were maintained in complete medium (RPMI-1640 supplemented with 10% FBS, glutamine and gentamycin) and cultured at 37 °C with 5% CO_2_. Viability following inhibitor treatment was monitored with CellTiter Glo.

### RNA and protein expression

RNA preparation is carried out using the Nucleospin RNA isolation kit (Macherey-Nagel, Düren, Germany). Following cDNA synthesis using iScript cDNA synthesis kit (Bio-Rad, Hercules, CA, USA) indicated genes were amplified using a SYBR green PCR mastermix (Kapa Biosystems, Woburn, MA, USA). Primer sequences are available on request. The ΔΔCT method was used to calculate the relative expression.

For western blot analysis of protein expression, cell pellets or tumor pieces lysed in lysis buffer as described before.^25^ A unit of 50 *μ*g of protein was resolved on 4–20% ClearPAGE gels (C.B.S Scientific Company, San Diego, CA, USA) and transferred to nitrocellulose membrane (Protran, GE Healthcare Bio-Sciences, Piscataway, NJ, USA). The membrane was blotted with specific antibodies. Antibodies against the following proteins were used: phosphorylated ATR (GeneTex, Inc., Irvine, CA, USA); cleaved PARP (Cell Signaling Technology, Danvers, MA, USA); ATR (Santa Crutz Biotechnology, Dallas, TX, USA); ATF4 (Santa Crutz Biotechnology); CHOP (Santa Crutz Biotechnology); phosphorylated H2Ax (*γ*H2Ax; Merck-Millipore), p62 (Progen Biotechnik, Heidelberg, Germany) and Actin (Sigma-Aldrich, St. Louis, MO, USA).

### Mouse experiments

All animal experiments were performed in accordance with EU directive 2010/63 (regional animal ethics committee of Gothenburg #36-2014). The PDXs were obtained by injecting 2 × 10^5^ cells mixed with equal volume of Matrigel (Corning, NY, USA) subcutaneously at the flank of immunocompromised, non-obese severe combined immune-deficient interleukin-2 chain receptor *γ* knockout mice (NOG mice; Taconic, Ry, Denmark) as described previously.^30^ Tumors were measured with caliper at regular time points and tumor volume calculated using the formula: tumor volume (mm^3^)=(length(mm)) × (width(mm))^2^/2. B16F10-luciferase cells were transplanted by subcutaneous injection. Seven days after transplantation, mice were injected with 100 *μ*l of 30 mg/ml d-luciferin. Mice were sedated in an isofluran administrating chamber and then placed in an IVIS Lumina III XR machine (Perkin-Elmer, Norwalk, CT, USA).

### Statistical analysis

Values are presented as mean±S.D. when data are combined. For statistical analyses, we used Graphpad Software, Inc. (La Jolla, CA, USA): multiple *t*-test or one-way ANOVA (with Sidak corrections) for tumor burden, and the log-rank test for survival. All mouse experiments contained 3–5 mice per group.
