# Supplementary material for: BET bromodomain inhibitors synergize with ATR inhibitors in melanoma
Source: Cell Death Dis. 2017 Aug 10;8(8):e2982–. doi: 10.1038/cddis.2017.383 (PMC5596569; doi:10.1038/cddis.2017.383)
Supplement: Supplementary Figures [file cddis2017383x1.pdf]

Figure S1

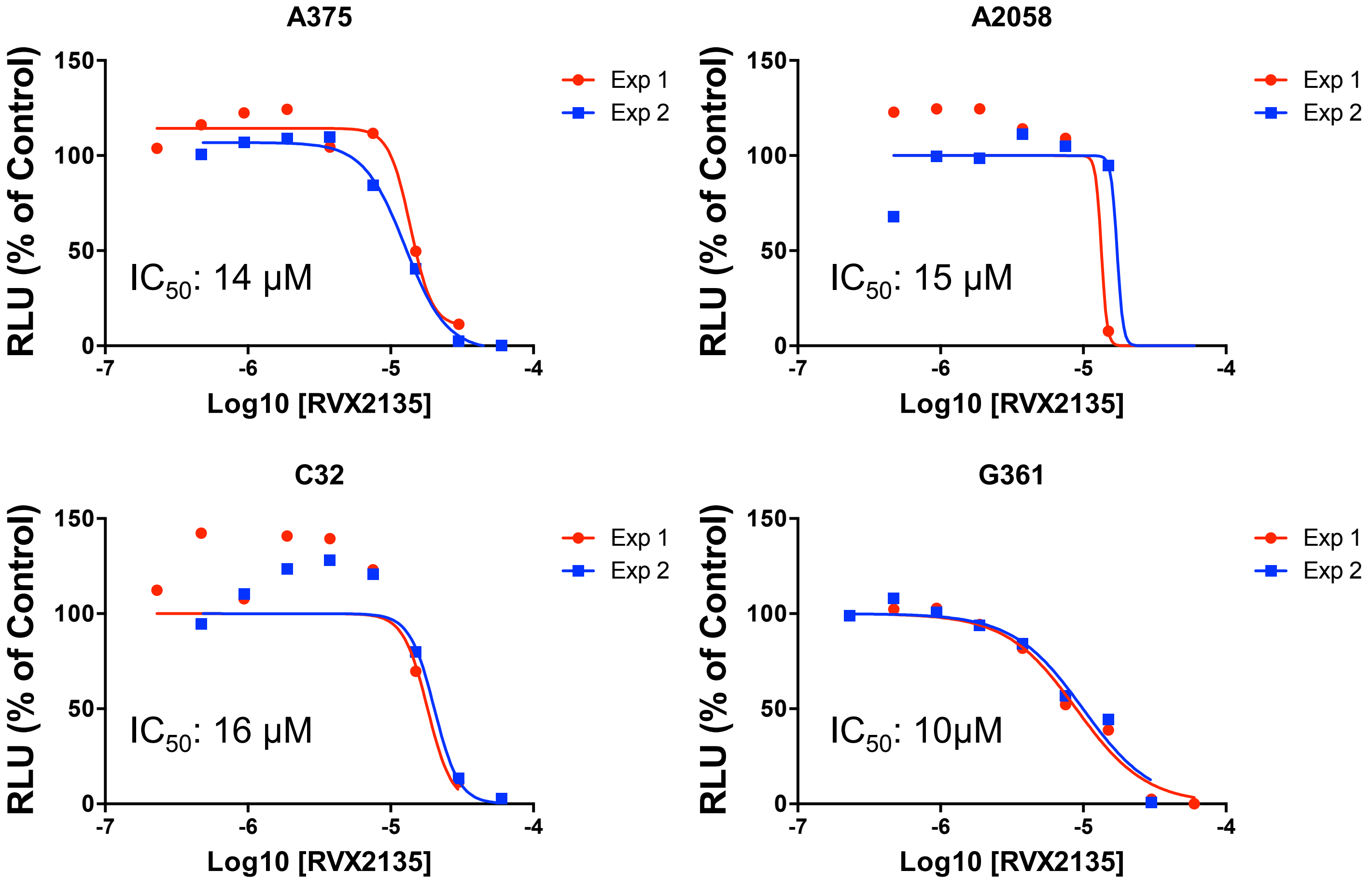

Figure S1. BETI RVX2135 inhibit melanoma cell proliferation. Melanoma cells were cultured in RPMI +10% FBS and antibiotics in the presence of increasing concentrations of RVX2135 for 48 h. CellTiterGlo (Promega) and a luminometer was used to measure viability. Shown are graphs of mean values in two independent experiments performed in triplicates.

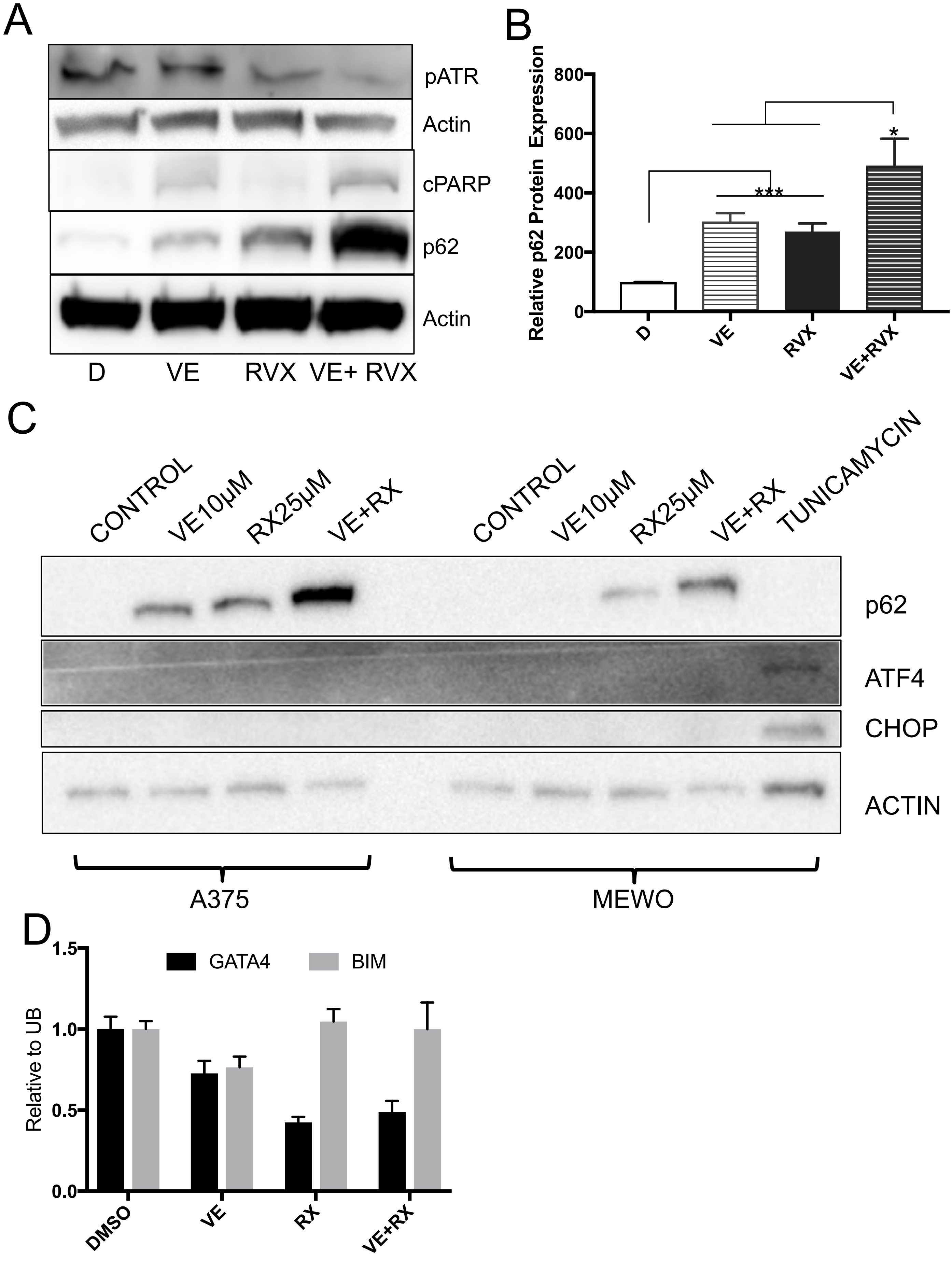

Figure S2. ATR inhibitors synergize with BETi to kill melanoma cells and induce SASP/ER stress. A) MeWo cells were treated with vehicle, VE821, RVX and were analyzed by Western blot analysis using indicated antibodies. B) Denistometric analysis of three different biological repeats. C) A375 and MeWo cells were treated as in A but also with tunicamycin, an ER stress inducer. D) A375 cells were treated as indicated and analyzed by qRT-PCR using primers against GATA4 and BIM.
